# Inductive heating kills cells that contribute to plaque: a proof-of-concept

**DOI:** 10.7717/peerj.929

**Published:** 2015-04-28

**Authors:** Angelo Gaitas, Gwangseong Kim

**Affiliations:** Kytaro Inc., Miami, FL, USA; Electrical and Computer Engineering Department, Florida International University (FIU), Miami, FL, USA

**Keywords:** Remote cell death, Microparticles, Atherosclerosis treatment methodologies, Electromagnetic induction heating, Translational research

## Abstract

Inducing cell death by heating targeted particles shows promise in cancer treatment. Here, we aim to demonstrate the feasibility of extending the use of this technique to treat and remove vascular deposits and thrombosis. We used induction heating of macrophages, which are key contributors to atherosclerosis and have demonstrated clear feasibility for heating and destroying these cells using ferromagnetic and pure iron particles. Specifically, iron particles achieved maximum temperatures of 51 ± 0.5 °C and spherical particles achieved a maximum temperature of 43.9 ± 0.2 °C (*N* = 6) after 30 min of inductive heating. Two days of subsequent observation demonstrated that inductive heating led to a significant reduction in cell number. Prior to induction heating, cell density was 105,000 ± 20,820 cells/ml (*N* = 3). This number was reduced to 6,666 ± 4,410 cells/ml for the spherical particles and 16,666 ± 9,280 cells/ml for the iron particles 24 h after inductive heating. Though cell density increased on the second day following inductive heating, the growth was minimal. Cells grew to 26,667 ± 6,670 cells/ml and 30,000 ± 15,280 cells/ml respectively. Compared to cell cultures with iron and spherical particles that were not subjected to induction heating, we observed a 97% reduction in cell count for the spherical particles and a 91% reduction for the iron particles after the first 24 h. After 48 h we observed a 95% reduction in cell growth for both spherical and iron particles. Induction heating of microparticles was thus highly effective in reducing the macrophage population and preventing their growth. These results demonstrate the feasibility of targeting cells involved in atherosclerosis and warrant further research into potential clinical applications.

## Introduction

Electromagnetic induction heating is the heating of an electrically conducting object (in this case microparticles) by an alternating magnetic field created by a high-frequency alternating current (AC) passing through a coil (Semiatin, 1988). Generated Eddy currents on and within the particles lead to Joule heating (Semiatin, 1988). Induction heating was first suggested as a means for therapy (Gilchrist et al., 1957) in 1957, and it has been proposed for cancer therapy to reduce the size of tumors by specifically targeting tumor cells with nanoparticles, which are then heated to high enough temperatures to cause death of cancerous cells (Jordan et al., 1997; Jordan et al., 1999; Van der Zee, 2002; CF Chan & B Kirpotin, 1993). Given the promising results for application of inductive heating in cancer therapeutics, we have aimed to demonstrate the feasibility for applying this method to other pathologies. Specifically, we have chosen to test the ability of inductive heating to destroy cells associated with arthrosclerosis and thrombosis, as prevention strategies and treatments for these conditions are limited in their effectiveness.

In atherosclerosis, chronic inflammation of the arterial wall is caused by the buildup of macrophages, white blood cells, platelets, and other particles that form plaque (Maton et al., 1993). Following plaque formation, stenosis and aneurysm occur, and plaque may eventually rupture and cause acute coronary events (Lusis, 2000). In thrombosis, a blood clot is formed inside a blood vessel made from platelets and fibrin (Furie & Furie, 2008). The clot obstructs blood flow, ultimately creating anoxia and tissue death. A clot may also break free becoming an embolus.

Current treatment methodologies for atherosclerosis include life style change, medication, and surgical interventions.^1^1http://www.nhlbi.nih.gov/health/health-topics/topics/atherosclerosis/treatment.html Life style changes and medications largely rely on delaying the progress of plaque buildup rather than removing it. The surgical interventions are limitedly performed for severe atherosclerosis cases (see footnote 1). Treatment for thrombosis mainly involves the use of anticoagulant medication. While anticoagulant therapy prevents worsening of thrombosis, it does not remove the thrombus and comes with various risks, including bleeding, recurrence of thrombosis, pulmonary embolism, and post-thrombotic syndromes. There is thus a great need for treatements that can reduce the pre-existing vascular buildups and restore the normal circulation while avoiding invasive surgical procedures.

Here we have employed a method of inductive heating of micrometer-sized particles to break up vascular deposits and thrombus, which are associated with a number of health complications,^2^2http://www.cdc.gov/heartdisease/facts.htm to test if this method can effectively destroy these problematic cells. Our results show significant reduction in cell number as a result of inductive heating and support the view that inductive heating as a therapeutic option should be more extensively studied.

## Materials and Methods

### Materials

In this preliminary work, we used spherical carboxyl ferromagnetic particles, prepared using chromium dioxide and coated onto uniform polystyrene particles, at a concentration of 0.5% w/v and diameter 28.0–34.9 µm (catalog # CFM-300-5 from Spherotech). We also used pure iron particles <44 µm (powder Fe-110 from Atlantic Equipment Engineers).

**Figure 1:**
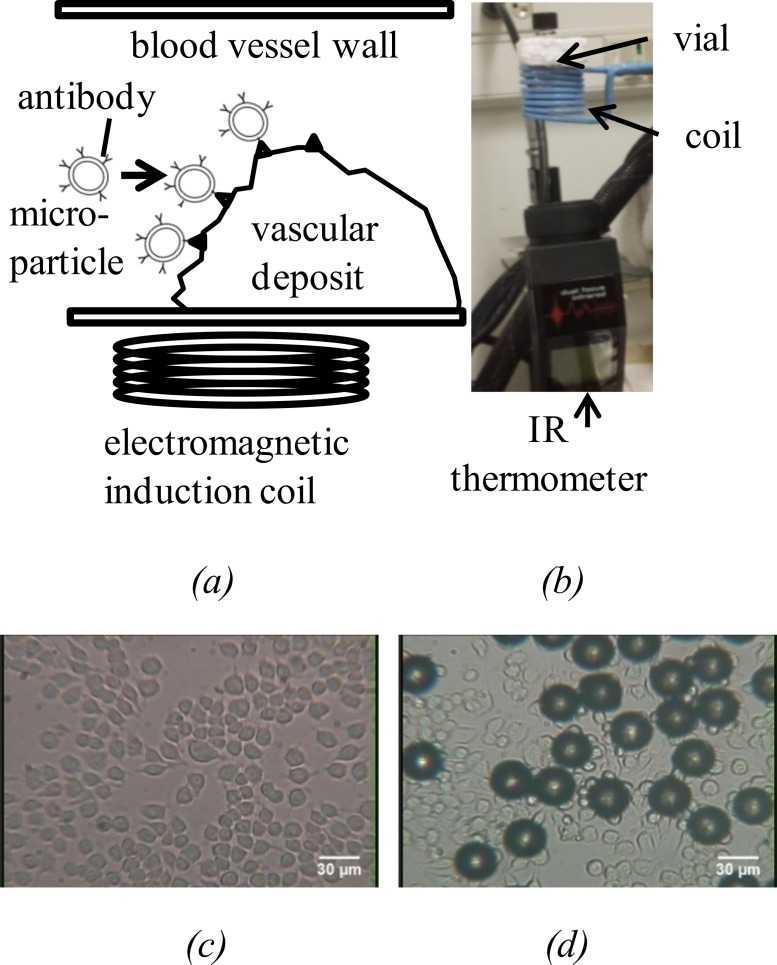
(A) is an illustration of micron-sized particles attached on part of vascular deposit site with at least one type of a biological binder, such as an antibody. Inductive heating is used to reduce vascular deposits by heating the targeted vascular deposits at relevant temperatures. (B) The set-up used to heat cells with the micro-particles. (C) Macrophages in vial. (D) Macrophages in vial with spherical particles (diameter 28.0–34.9 µm) attached after 2 h of incubation (×200 magnification).

An induction heating unit was used externally to create an alternating magnetic field strong enough to heat those particles and reduce cell buildup (as shown conceptually in Fig. 1A). Micro- and nano- particles have been extensively studied as carrier devices to deliver drugs or functional materials to target sites and release/actuate the payload in a controlled manner (Rabinow, 2004; Reddy et al., 2006; Kumar, 2000; Brannon-Peppas & Blanchette, 2012; Birnbaum & Brannon-Peppas, 2004; Torchilin, 2011).

Nanoparticles are widely used for targeted delivery approaches based on their ability to penetrate through the vascular wall to deeper tissue, the ease with which cells uptake them, and the minimal physical disturbance they cause cells due to their smaller size. However, microparticles have been used more frequently for controlled release applications based on biodegradation. It was recently reported that micrometer sized particles can bind more efficiently to vascular walls than nanometer sized particles can (Charoenphol et al., 2011; Charoenphol et al., 2012), making micro-size particles more appropriate for our application.

### Cell culture

We chose macrophages as a model cell line that represents the atherosclerotic condition. Macrophages are the main component of arterial plaque along with platelets and white blood cells. Macrophages play a central role in atherosclerosis, including scavenging of modified lipoprotein, generation of foam cells, breaking down/thinning of the endothelial layer (fibrous cap) to turn into unstable lesions, secretion of cytotoxic molecules to promote death of surrounding cells, forming necrotic core, and so on (Moore & Tabas, 2011; Schrijvers et al., 2007; Dickhout, Basseri & Austin, 2008). Reduction of macrophages can help reduce atherosclerosis (Croons, Martinet & RY De Meyer, 2010; Saha et al., 2009; Tabas, 2005). A number of recent studies suggested using macrophage as therapeutic target for artherosclerosis (Glass, 2002; Demidova & Hamblin, 2004; Amirbekian et al., 2007). In addition, macrophages are typical phagocytotic cells. In the immune system macrophages ingest pathogenic microorganisms and have the ability to bind randomly to foreign particles including the micro-particles we introduced. The non-specific phagocytosis process was utilized to simulate cell binding to microparticles. The microparticles used in this study were not modified for targeting.

Our inductive heating was performed on a murine macrophage cell line, RAW 264.7, These macrophages (Fig. 1C) were cultured in Dulbecco’s Modified Eagle Medium (DMEM) and supplemented with 10% fetal bovine serum and 1% Pen-Strep. For the induction experiments, cells were seeded in 4 mL glass vials (40,000 cells) instead of in a culture flask.

### Cell death by heating

To determine the temperature required to achieve necrosis of cells, we placed the vials on a temperature controlled heating plate (Lakeshore temperature controller with 0.1 K accuracy). The temperature was measured with both a thermocouple (Omega, Stamford, Connecticut, USA) in contact and an infrared thermometer (Infrared Thermometer Dual Focus LS; Micro-Epsilon, Raleigh, North Carolina, USA) remotely. The vials were kept in an incubator overnight to allow surviving cells to grow. The following day, macrophage cells were lifted by trypsinization at room temperature, and vigorously flushed. The number of cells in suspension was determined by hemocytometry.

### Cell death by induction heating

Macrophage cells were prepared in the 4 mL vials (40,000 cells/mL input). The following day, microparticles were added into the cell solution at 0.1 mg/mL final concentration and incubated for a minimum of 2 h to allow for cells to attach (Fig. 1D). The vials were placed in the center of the induction coil. Induction heating was generated using an Easyheat induction heating system (Ambrell-Ameritherm Company, Scottsville, New York, USA). A picture of the set-up is shown in Fig. 1B. An infrared thermometer was placed under the vial to measure the temperature of the cells and the microparticles. The frequency used was 350 kHz, the current through the coil was 178.5 A, and the power was 1 kW, which provided a field intensity of 34 kA/m at the center of the coil. After completing the heating procedure, the cells were kept in an incubator (37 C, 5% CO_2_, and nearly saturated moisture) overnight. The following day, the cells were detached from the glass bottom by trypsinization at room temperature and by vigorous flushing with a pipette. The cells were mixed with the equivalent volume of trypan blue solution to discriminate live cells from dead ones and counted with a hemocytometer using an Olympus IMT-2 Inverted Phase Contrast Fluorescence microscope (Olympus, Shinjuku, Tokyo, Japan). The process was repeated a day later with the remaining vials.

In addition to our test group, we employed two control groups. A batch of cells, which were targeted with particles that did not undergo inductive heating served as one control, to ensure that the particles themselves were not cause for cell death through, for example, toxicity. We also used a batch of cells without targeted particles as a separate control. We inductively heated 6 vials from each of our test group on day zero. After 24 h, we counted cells from half of the vials from each group, and following another 24 h, we counted cells from the remaining vials.

## Results

Using our induction heating process (Fig. 1), we first tested what temperature was required to achieve necrosis of cells. To do this, we placed vials on temperature-controlled heating plates, and we set the temperature to 37 °C, 45 °C and 55 °C for 30 min each. After 24 h, we found that there were about 40,000 cells/ml at a temperature of 37 °C, 14,000 cells/ml heated to 45 °C and no cells survived (0 cells/ml) at temperatures of 55 °C.

**Figure 2:**
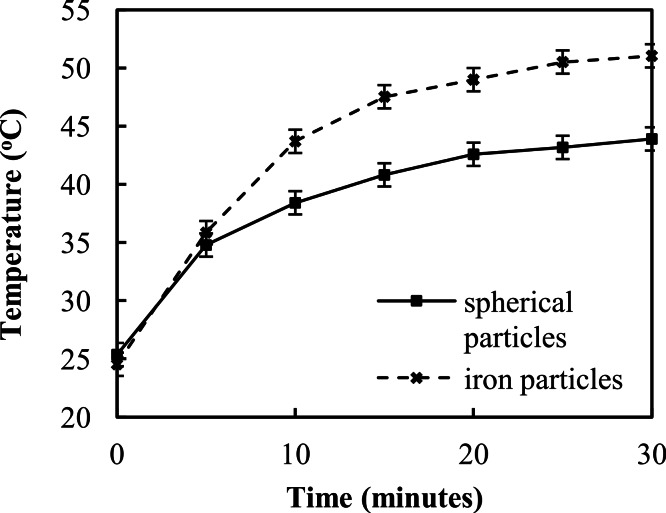
Temperature change over time of particles on cells in vials and in the alternating magnetic field with standard error bars (sample number *N* = 6).

Induction heating results of the two particle types on cell culture in media are shown in Fig. 2. The iron microparticles achieved a maximum temperature of 51 ± 0.5 °C after 30 min (*N* = 6), while the spherical particles achieved a maximum temperature of 43.9 ± 0.2 °C (*N* = 6). It should be noted that these values represent the temperature of the entire solution. Heating by induction is spatially confined within the short vicinity of particles. Therefore, the effective temperature at the particle may be much higher than the bulk temperature measured by the IR thermometer.

**Figure 3:**
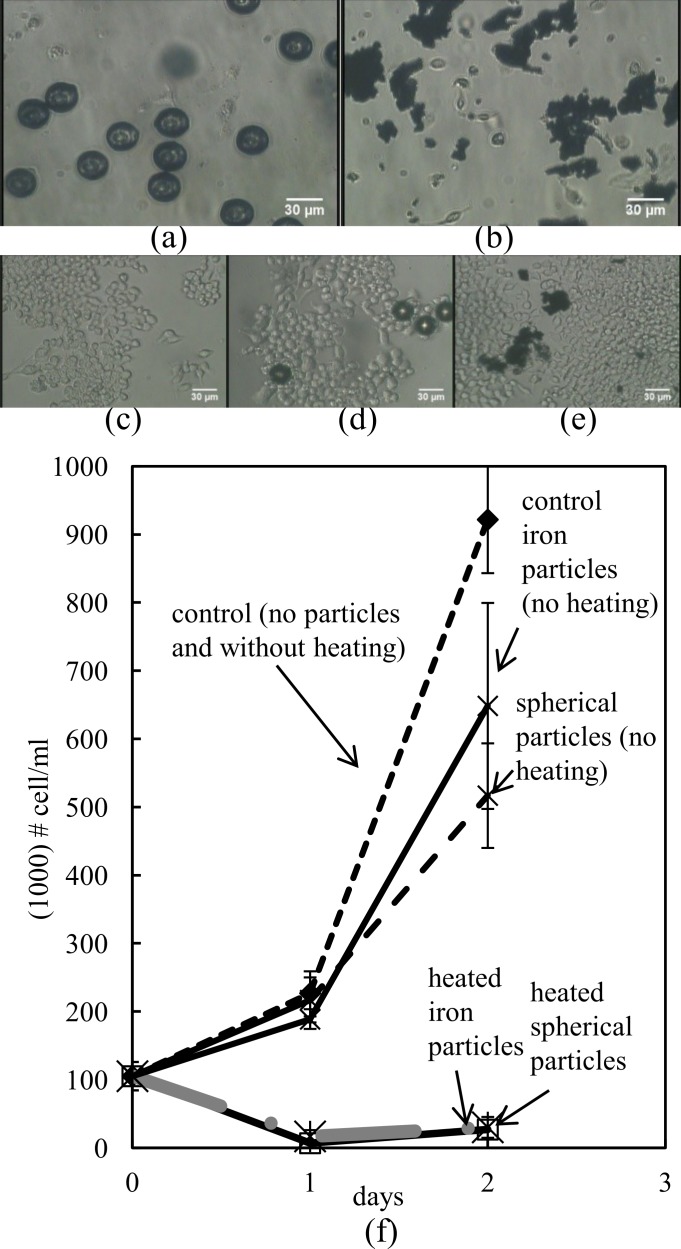
(A) Heated spherical particles on cells on day 2 (mag. ×200). (B) Heated iron particles on cells on day 2 (mag. ×200). (C) Control vial on day 2 (mag. ×200). (D) Spherical particles on cells in control vials on day 2 (mag. ×200). (E) Iron particles on cells in control vials on day 2 (mag. ×200). (F) Hemocytometry cell count of cell growth. Results with standard error of mean (*N* = 3).

We investigated the differences in cell number across groups as a way to determine the actual impact of inductive heating on cell death. Significant reduction in cell survival was observed in inductively heated cells, compared to the controls, as shown in Fig. 3. While the initial number of cells was 105,000 ± 20,820 cells/ml, 24 h after induction heating the cell count was reduced to 6,666 ± 4,410 cells/ml for the spherical particles and to 16,666 ± 9,280 cells/ml for the iron particles (*N* = 3). After an additional 24 h, the cell count was 26,667 ± 6,670 cells/ml for the spherical particles and 30,000 ± 15,280 cells/ml for the iron particles respectively (*N* = 3). In contrast, the cells that with no particles grew to 226,670 ± 23,330 cells/ml after 24 h and to 921,670 ± 78,550 cells/ml after 48 h. Further, the cells with spherical particles that were not subjected to heating grew to 216,660 ± 42,262 cells/ml after 24 h and 516,660 ± 76,720 cells/ml after 48 h. Cells with iron particles that were not heated grew to 188,333 ± 4,409 cells/ml after 24 h and to 648,333 ± 151,116 cells/ml after 48 h. (*P* < 0.002 of controls vs. ferromag. day 1 and iron day 1, *t*-test. *P* < 0.001 of controls vs. ferromag. day 2 and iron day 2, *t*-test.)

## Discussion

The present study demonstrates that induction heating of two types of microparticles can feasibly cause effective damage to macrophage cells, which is a cell line that is highly relevant to atherosclerosis. Though induction heating has been primarily investigated in the context of cancer therapy, we believe that its application to cancer should be extended and that we should begin investigating the potential to use induction heating to treat vascular buildups. These results can help inform therapeutic parameters as translational research is pursued, including the effective particle size, type of particles, and possible temperature range that could be effective for destroying macrophages in atherosclerosis.

Our findings demonstrate that spherical particles have a better ability to bind to cells than random shaped iron particles. While most of the spherical magnetic particles attached to cellular surfaces, iron particles were only partially uptaken by macrophage cells. The highest temperature achieved by spherical magnetic particles was ∼43 °C, but cell death at this temperature was greater than that achieved by the iron particles, which were heated at higher temperatures (∼51 °C).

Our study included some limitations. For instance, we observed that cells without microparticles exhibited higher growth compared to the controls that included microparticles but were not subjected to heating. This reduction in cell growth may be due to the activation of the phagocytotic activity of the cells rather than the toxicity of the particles, because the exponential growth pattern was still observed.

The heating of metal particles by induction was confined to the narrow vicinity around the particle. The temperature in the local region could therefore be much higher than bulk temperature we measured. The tighter contact of cells with the spherical microparticles may explain the results. Ultimately, we believe that the local temperature should be high enough to cause cell lysis, not just necrosis because these temperatures would ensure reduction of the plaque or thrombus, while avoiding creating an embolus.

Future research should address the several challenges related to treating plaque build-up in atherosclerosis and thrombosis with heat induction. For example, heating can cause platelet aggregation, leading to an inflammatory response or clotting. This issue could potentially be addressed by fabricating microparticles whose half surface that does not stick to organic materials such as platelets, while the other half is coated with target antibodies. Determining the ideal number and size of microparticles to use during heat induction should also be investigated in future studies.

Eventually, surface functionalization of particles will be necessary to selectively deliver the particles to arterial plaque. A biological targeting moiety (antibodies, peptides, aptamers etc.) attached to the particles that will selectively bind to one of the substances that makes up vascular deposits would be required when flow is introduced. The methodology for specific targeting to arterial plaque is not well established yet. The microparticles can, for instance, be coated with more than one binder, mimicking monocytes which initiate plaque buildup. In one example, one antibody may aid in rolling adhesion (selectin) and another antibody can be used for stationary adhesion (integrin).

An advantage of induction heating is that the therapeutic efficacy can be activated only where the magnetic field is applied. This localization could minimize the possible side effects of non-specific uptake of particles by other distant organs. Thus the accumulation of particles elsewhere may not be damaging because they will not be heated.

In this introductory work we used commercially available particles. However, microparticles can also be manufactured with microfabrication methods such as surface micromachining to control their size, dimensions, properties and function. The magnetic particles can also be used in combination with (or as part of) a magnetic resonance imaging (MRI) contrast agents for monitoring the vascular deposits with MRI contrast enhancement while the patient is undergoing treatment (Ruehm et al., 2001; Yu et al., 2000).

In a real life setting microparticle extraction would be necessary. The particles can be extracted from a patient by placing a magnet in proximity to the area of extraction causing the particles to flow out of a blood vessel. Alternatively, the microparticles can be fabricated with biodegradable materials containing magnetic payloads to facilitate clearance from the body. Thus, there are a number of options and opportunities related to the development of techniques using heating induction with microparticles to treat atherosclerosis and thrombosis specifically, as well as other diseases.

## Conclusions

In this manuscript, we have demonstrated the feasibility of destroying macrophages, which contribute to the plaque associated with arthrosclerosis and thrombosis, using inductively heated micrometer-sized particles. The results indicate that the technique was highly effective in reducing a cell culture population. We have attempted to introduce the concept and possibility of using induction heating in conjunction with microparticles for therapeutic applications in medicine. Though much research is required before application of this technique could be clinically implemented, our study demonstrates the potential value of the technique and suggests that further research in this area could provide clinical benefits.

## Supplemental Information

10.7717/peerj.929/supp-1 File S1 Supplemental File
