# Supplementary material for: Inductive heating kills cells that contribute to plaque: a proof-of-concept
Source: PeerJ. 2015 Apr 28;3:e929. doi: 10.7717/peerj.929 (PMC4419522; doi:10.7717/peerj.929)
Supplement: File S1 [file peerj-03-929-s001.docx]

**Supplemental File**

Raw data for Figure 2

Spherical Particles

|  | Temperature (C) | | | | | | | |
| --- | --- | --- | --- | --- | --- | --- | --- | --- |
| Time (Min) | Vial 1 | Vial 2 | Vial 3 | Vial 4 | Vial 5 | Vial 6 | Average | Standard Error |
| 0 | 25 | 27 | 25 | 21.3 | 28.8 | 25.1 | 25.36 | 1.02 |
| 5 | 36.4 | 36 | 36 | 31.8 | 36.2 | 32.3 | 34.78 | 0.86 |
| 10 | 37.9 | 40 | 40.7 | 34 | 40 | 37.9 | 38.41 | 1 |
| 15 | 40.1 | 42.6 | 42 | 38.3 | 41.8 | 40.1 | 40.81 | 0.65 |
| 20 | 42.1 | 44.1 | 42.8 | 41.9 | 42.8 | 41.8 | 42.58 | 0.35 |
| 25 | 43.05 | 43.3 | 43 | 43.1 | 43.5 | 43.1 | 43.17 | 0.07 |
| 30 | 43.1 | 43.93 | 44.7 | 44 | 44 | 43.7 | 43.9 | 0.21 |

Iron Particles

|  | Temperature (C) | | | | | | | |
| --- | --- | --- | --- | --- | --- | --- | --- | --- |
| Time (Min) | Vial 1 | Vial 2 | Vial 3 | Vial 4 | Vial 5 | Vial 6 | Average | Standard Error |
| 0 | 18.7 | 25.4 | 24.3 | 28 | 24.3 | 26.5 | 24.53 | 1.3 |
| 5 | 34.5 | 35.2 | 35.2 | 35.5 | 35.2 | 39.5 | 35.85 | 0.74 |
| 10 | 44.3 | 41.4 | 42.5 | 44.7 | 43.3 | 46 | 43.7 | 0.67 |
| 15 | 48.1 | 48.5 | 47.1 | 49.1 | 43.9 | 48.5 | 47.53 | 0.77 |
| 20 | 49.1 | 50.8 | 49.5 | 51.1 | 44 | 49.5 | 49 | 1.05 |
| 25 | 51.4 | 51.4 | 50.5 | 51.8 | 48 | 50 | 50.51 | 0.57 |
| 30 | 51.6 | 51.8 | 51 | 51.8 | 48.7 | 51.4 | 51.05 | 0.48 |

Raw Data For Figure 3

Heated Spherical Particles

| Day 1 | Day 1 | Day 2 |
| --- | --- | --- |
| Vial 1 | 0 | 20000 |
| Vial 2 | 5000 | 20000 |
| Vial 3 | 15000 | 40000 |
| Average | 6667 | 26667 |
| Standard Error | 4410 | 6667 |

Heated Iron Particles Cell Count

|  | Day 1 | Day 2 |
| --- | --- | --- |
| Vial 1 | 35000 | 60000 |
| Vial 2 | 10000 | 20000 |
| Vial 3 | 5000 | 10000 |
| Average | 16667 | 30000 |
| Standard Error | 9280 | 15275 |

Control (No Particles And Without Heating) Cell Count

|  | Day 0 | Day 1 | Day 2 |
| --- | --- | --- | --- |
| Vial 1 | 145000 | 220000 | 990000 |
| Vial 2 | 95000 | 270000 | 1010000 |
| Vial 3 | 75000 | 190000 | 765000 |
| Average | 105000 | 226667 | 921667 |
| Standard Error | 20817 | 23333 | 78546 |

Control Spherical Particles (No Heating) Cell Count

|  | Day 1 | Day 2 |
| --- | --- | --- |
| Vial 1 | 205000 | 435000 |
| Vial 2 | 295000 | 670000 |
| Vial 3 | 150000 | 445000 |
| Average | 216667 | 516667 |
| Standard Error | 42262 | 76721 |

Control Iron Particles (No Heating) Cell Count

|  | Day 1 | Day 2 |
| --- | --- | --- |
| Vial 1 | 190000 | 550000 |
| Vial 2 | 195000 | 945000 |
| Vial 3 | 180000 | 450000 |
| Average | 188333 | 648333 |
| Standard Error | 4410 | 151116 |

We used GraphPad QuickCalcs (www.graphpad.com) for the statistical analysis.
